# Characterization and Manipulation of the Crosstalk Between Dendritic and Natural Killer Cells Within the Tumor Microenvironment

**DOI:** 10.3389/fimmu.2021.670540

**Published:** 2021-05-14

**Authors:** Benedikt Jacobs, Veronika Gebel, Lukas Heger, Victoria Grèze, Hansjörg Schild, Diana Dudziak, Evelyn Ullrich

**Affiliations:** ^1^ Department of Internal Medicine 5, Haematology and Oncology, Friedrich Alexander University Erlangen-Nuremberg (FAU), University Hospital Erlangen, Erlangen, Germany; ^2^ Children’s Hospital, Goethe-University Frankfurt, Frankfurt, Germany; ^3^ Experimental Immunology, Goethe University Frankfurt , Frankfurt, Germany; ^4^ Frankfurt Cancer Institute, Goethe University, Frankfurt, Germany; ^5^ Department of Dermatology, Laboratory of Dendritic Cell Biology, University Hospital Erlangen and Friedrich-Alexander University Erlangen-Nuremberg (FAU), Erlangen, Germany; ^6^ Institute of Immunology, University Medical Center Mainz, Mainz, Germany; ^7^ Research Centre for Immunotherapy, University Medical Center Mainz, Mainz, Germany

**Keywords:** NK cells, dendritic cells, DC-NK cell interaction, tumor microenvironment, cellular therapies

## Abstract

Cellular therapy has entered the daily clinical life with the approval of CAR T cell therapeutics and dendritic cell (DCs) vaccines in the US and the EU. In addition, numerous other adoptive cellular products, including natural killer (NK) cells, are currently evaluated in early phase I/ II clinical trials for the treatment of cancer patients. Despite these promising accomplishments, various challenges remain to be mastered in order to ensure sustained therapeutic success. These include the identification of strategies by which tumor cells escape the immune system or establish an immunosuppressive tumor microenvironment (TME). As part of the innate immune system, DCs and NK cells are both present within the TME of various tumor entities. While NK cells are well known for their intrinsic anti-tumor activity by their cytotoxicity capacities and the secretion of pro-inflammatory cytokines, the role of DCs within the TME is a double-edged sword as different DC subsets have been described with either tumor-promoting or -inhibiting characteristics. In this review, we will discuss recent findings on the interaction of DCs and NK cells under physiological conditions and within the TME. One focus is the crosstalk of various DC subsets with NK cells and their impact on the progression or inhibition of tumor growth. In addition, we will provide suggestions to overcome the immunosuppressive outcome of the interaction of DCs and NK cells within the TME.

## Introduction

Tumor surveillance is achieved by a complex interplay of the components of the innate and adaptive immune system. Here, we summarize our current knowledge on the role and interaction of dendritic cells (DCs) and natural killer (NK) cells in the tumor microenvironment (TME). While circulating NK cells are efficient at identifying and eliminating tumor cells, DCs bridge the innate and adaptive immune system via the uptake of tumor cell debris and the subsequent presentation of tumor-specific antigens to T cells (1). NK cells and DCs are currently used for immunotherapies to treat tumor patients, for NK cells exhibit the ability to directly eliminate tumor cells without prior sensitization, while DCs are able to initiate an immune response by presenting antigens and inducing tumor-antigen specific CD8^+^ T cell (2). Moreover, when DCs and NK cells encounter each other, they are able to promote each other's activation, maturation, and functional activity (3). Within the TME, conventional type 1 DCs (cDC1s) show a bidirectional crosstalk with NK cells, increasing the selective recruitment of cDC1 together with their differentiation, and maturation as well as NK cell activation. This allows a better tumor control, patient survival and improved therapeutic responses (4, 5). However, it is known that tumor cells develop highly efficient mechanisms to impair the functionality of NK cells and DCs to escape immune surveillance in order to ensure their own survival. The tumor establishes an immune suppressive environment by the secretion of immune suppressive cytokines and chemokines, by metabolic acidification (6), by the recruitment of immune-regulatory cells, such as regulatory T cells (T regs) and myeloid-derived suppressor cells (MDSCs), by affecting the polarization of macrophages as well as by up-regulation of immunosuppressive proteins including ligands for immune checkpoint (ICP) receptors, such as PD-L1. The latter have the ability to suppress the anti-tumor activity of DCs and NK cells as well as their ability to promote each other. Moreover, DCs may even switch from a tumor-inhibiting to a tumor-promoting subtype within the TME (7). In order to overcome the suppressive effect of the TME on NK cells and DCs, the bidirectional crosstalk of NK cells and DC1 plays a very important role in the coordination of immune responses against cancer. That is why the presence of both DC1s and NK cells within the TME is primordial, and their interaction represents a potential target to improve the efficacy of cancer immunotherapy (2).

In this review, we will first give a short overview about the biology of DCs and NK cells. Next, we will describe their interaction under physiological conditions and within the TME. Finally, we will discuss possibilities to overcome the suppressive impact of the TME and to restore efficient tumor immune surveillance.

## NK Cell Biology

As part of the innate immune system, NK cells have the ability to lyse virally infected and malignant cells. They play an important role at eradicating cancer cells and in shaping the response of adaptive immune cells. NK cells develop and differentiate in the bone marrow and represent approximately 10% of the lymphocytes in the peripheral blood (PB). NK cells express CD56 on their surface and lack CD3 expression (CD56^+^CD3^−^). Human NK cells are separated into two subsets, with an immune regulatory CD56^bright^CD16^−^ population harboring potent cytokine production capability and a highly cytotoxic CD56^dim^CD16^+^ population, representing the major NK cell population within the peripheral blood (90%). CD56^dim^CD16^+^ express the Fc gamma receptor III CD16 recognizing the Fc portion of antigen-bound antibodies allowing them to lyse the opsonized cell, a mechanism called antibody dependent cell-mediated cytotoxicity (ADCC) (8).

In contrast to T or B cells, NK cells recognize their target cells in an antigen-independent manner regulated by a variety of activating and inhibiting receptors. NK cell cytotoxicity is regulated by balanced signals of these activating and inhibitory receptors (9, 10).

Major histocompatibility (MHC) class I molecules, expressed on all healthy nucleated cells, act as ligands for the killer-immunoglobulin-like receptor-group (KIR) or natural killer group 2A (NKG2A), which are mainly responsible for providing NK cell inhibiting signals. These mechanisms ensure that NK cells become selectively activated by cells that lack MHC-I-expression, such as tumor cells, but tolerate healthy tissue in their immune response, the so called “missing-self”-theory. If MHC-I expression is absent, the inhibitory receptor signal is missing, and NK cells can be activated (11). Usually MHC-I-expression is reduced or completely down-regulated on virally infected or malignantly transformed cells as an escape mechanism from cytotoxic T cell recognition, which on the other hand renders them sensitive to NK cell killing (8, 12).

The most important signals for activation of NK cells are mediated by the family of the natural cytotoxicity receptors (NCRs), the C-type lectin-like receptor NKG2D and also certain KIR-subtypes. NKp30, NKp44 and NKp46 that belong to the group of NCRs are expressed on all activated NK cells and bind non-HLA-specific ligands like virus-derived molecules (HA/HN for NKp46, NKp44) or intracellular proteins (BAT3/BAG6 for NKp30), which are released following cell stress or transformation (13). Furthermore, MICA-, MICB- and ULPB (or RAETI)-encoded proteins bind to the NKG2D-receptor with their MHC-I-like extracellular domain. Those encoded proteins belong to a group of at least 100 different ligands which are selectively expressed on non-healthy cells for the induction of NK cell activation (14).

NK cell killing includes a variety of mechanisms. Upon engagement an immunological synapse is established between NK cells and their target cells. Subsequently, they release the content of their cytotoxic granules, including perforin and granzyme b, into the synaptic gap. While perforin penetrates the target cell’s membrane, granzyme b gets inside the cell and induce programmed cell death (2, 8). Moreover, NK cells are able to induce apoptotic cell death via expression of death receptor ligands like FasL and TNF-related apoptosis-inducing ligand (TRAIL) on their surface (8, 12).

## DC Biology

DCs are important regulators of immune responses (15). They are sentinels of the immune system and settle all lymphoid as well as peripheral tissues. Here, they constantly take up antigens from the surrounding and process these antigens. After migration to the local draining lymph node, they present these processed antigens as peptide MHC complexes to T cells. Depending on the environment during encounter of the antigen, DCs either induce peripheral tolerance (steady state) or T cell immunity (inflammation) (16–18). In steady state, DCs induce peripheral tolerance by presenting antigens without costimulatory molecules leading to anergy or the deletion of antigen-specific T cells. However, in inflammatory settings and instructed by microbiota-derived cues resulting in tonic IFNAR signaling DCs can be activated by danger signals or pathogens via pattern recognition receptors such as Toll-like receptors (TLR) or C-type lectin receptors (CLR) (1, 19, 20). This leads to enhanced expression of peptide MHC complexes as well as costimulatory molecules (e.g. CD40, CD86) and the secretion of T cell polarizing cytokines such as IL-12 (17). Thereby, DCs are capable to induce and direct T cell responses. However, DCs are also able to interact with cells of the innate immune system such as NK cells (4, 21–24).

As all immune cells, except for yolk sac-derived macrophages, including Langerhans cells, DCs originate from hematopoietic stem cells (HSC) in the bone marrow (25). After differentiation of HSCs into common myeloid progenitor (MPP) cells via the steps of multipotent progenitor (MPP) and lymphoid-primed multipotent progenitor (LMPP) cells, the common DC progenitor (CDP) separates from the common monocyte progenitor (cMoP). The CDP can give rise to all DC subsets including plasmacytoid (pDC) as well as conventional DCs (cDC). Depending on the expression of specific transcription factors such as IRF8 and IRF4, cDCs further differentiate in cDC1 and cDC2, respectively (18). In contrast to cDCs, pDCs can also arise from a common lymphoid progenitor (CLP). Terminal-differentiated as well as preDCs are able to leave the bone marrow and settle all lymphoid as well as peripheral tissues.

Based on ontogeny, surface receptor expression as well as functions, DCs are subdivided into different subsets, which are mainly conserved between human and mice (17, 18, 25–27). pDCs share high transcriptional similarities between humans and mice and are identified by the expression of Siglec-H, CD45R (B220), and CD317 (PDCA-1) in mice, and by CD303a (BDCA-2), CD304 (BDCA-4) as well as CD123 (IL-3Rα chain) in humans. In both species, they share the expression of TLR7 and TLR9 and strongly react to viral infections with the secretion of vast amounts of type I IFN. However, their true role in T cell stimulation is a matter of ongoing discussions (28–31).

cDCs can be further separated into cDC1 and cDC2 in both, mice, and humans. Human and murine cDC1 share the specific expression of CLEC9A, XCR1, and CADM1 (17, 32–36). While murine cDC1 further express CD8α in lymphoid and CD103 in non-lymphoid tissues, human cDC1 are identified by a high expression of CD141 (BDCA-3) (1). In both species, cDC1 highly express TLR3 and respond to stimulation of TLR3 with production of type III IFN (37). They further produce IL-12, which is crucial for interaction with T cells as well as with NK cells. In mice, cDC1 are the only cross-presenting DC subpopulation for the activation of cytotoxic CD8^+^ T cells. This highlights their vital role in the immune response in the context of tumors as well as viral infections (4, 38–40). Furthermore, they polarize CD4^+^ T cells preferentially into Th1 cells. However, recent studies on human DCs suggest that all DC subpopulations are able to cross-present antigens and cDC1 excel only in the cross-presentation of necrotic cell-derived antigens, presumably due to the expression of the F-actin receptor CLEC9A (41–44).

Human and murine cDC2 transcriptionally rely on IRF4 and share the expression of CD11c and SIRPα (18). Human cDC2 can be identified by the expression of CD1c (BDCA-1), CD301 (CLEC10A) as well as the FcϵR1α, while murine cDC2 express CD11b as well as CD4 and DCIR2 in lymphoid tissues (27, 45, 46). In general, cDC2 are a more heterogeneous DC subpopulation than cDC1. Recent approaches, using single cell transcriptomics, showed that both human and murine cDC2 harbor different subpopulations termed DC2/DC3 and cDC2a/b, respectively (25, 31, 46–49). In the murine system, cDC2 have a lower capacity to cross-present antigens to CD8^+^ T cells, however they are efficient to polarize naïve T cells into Th2 and Th17 cells (50–52). In contrast, in comparison to human cDC1, human cDC2 have been suggested to harbor similar capacity to cross-present antigens to CD8^+^ T cells. Furthermore, they have been shown to polarize CD4^+^ T cells into Th1 cells, which can be linked to the ability of human cDC2 to secrete IL-12p70 under inflammatory conditions (27). Both, cDC1 and cDC2 have a detrimental role in the preservation of peripheral tolerance as murine cDC1 convert naïve CD4^+^ T cells into FoxP3^+^ T reg cells, whereas cDC2 expand already existing FoxP3^+^ T reg cells in the periphery (53).

## NK-DC Interaction Under Physiological Conditions

The first key report on the interaction between DCs and NK cells to control tumor growth was published in 1999, describing the coordinated control of mesothelioma tumors in mice by both subsets (54). Since then, several groups have explored their complex relationship revealing a bidirectional crosstalk between DCs and NK cells. Human NK cells are able to induce DC maturation and IL-12 production upon co-culture with immature monocyte-derived DCs (moDCs), which was suggested to be contact-dependent and relies on the secretion of NK cell-derived cytokines like TNF-α (55, 56). In contrast, freshly isolated human NK cells are activated by lipopolysaccharide (LPS)-treated mature moDCs (55). NK cell activation by moDCs was shown to be mediated mainly by cytokines of the IL-12 family. Initial reports demonstrated that IL-12 secretion by LPS-matured human moDCs stimulates IFN-γ production in NK cells (57), and similar results have been reported for other members of this cytokine family. Further, IL-23 has been suggested to directly stimulate IFN-γ production in human CD56^bright^ NK cells, and in cooperation with IL-18, in CD56^bright^ and CD56^dim^ NK cells (58). Additionally, LPS-matured moDC-derived IL-27 increased IFN-γ production and upregulation of NKp46- and ADCC-dependent NK cell cytotoxicity (59). The importance of DC-derived IL-12 for NK cell activation has been illustrated within an *in vivo* model of mouse cytomegalovirus (MCMV) infection. Upon MCMV infection cDCs secrete IL-12p40 and IL-15 in a TLR9/MyD88-dependent manner leading to NK cell activation and IFN-γ production which ultimately results in MCMV clearance (60).

Moreover, other cytokines such as IL-15 or IFN-α were able to increase NK cell proliferation and survival as well as cytotoxicity, respectively (21, 61–64). Both cytokines are involved within the NK cell-mediated rejection of CD8^+^ T cell-resistant tumors within various tumor mouse models upon treatment with the STING agonist cyclic dinucleotide (CDN). CDN activated NK cells directly via type I IFN signaling and indirectly via IL-15Rα up-regulation in DCs, which also depended on type I IFN (65). Moreover, co-incubation of human peripheral blood NK cells with autologous lung DCs from smokers suffering from chronic obstructive pulmonary disease (COPD) resulted in an IL-15-dependent increased killing of epithelial lungs cells compared to DCs from smokers without COPD. Similar results were obtained when using lung DCs from mice who were exposed to cigarette smoke compared to pure air exposed ones (66).

In addition, activated NK cells were reported to specifically kill immature moDCs (56) via NKp30 (67) and DNAM-1 (68). This process was executed mainly by NKG2A^+^KIR^-^ NK cells. This observation can be explained due to the week expression of the inhibitory NKG2A receptor ligand HLA-E on immature moDCs compared to LPS-matured moDCs, whereas HLA class I molecules, the ligands for the inhibitory KIRs, are expressed on both DCs populations (69). Similar, NK cells have been shown to interfere with the induction of Th2-biased T cell responses by killing Th2 immune response inducing moDCs in a NKp30 and DNAM-1 dependent manner (70).

Interestingly, apart from its involvement in the killing of immature moDCs, engagement of NKp30 resulted in increased secretion of TNF-α and IFN-γ by NK cells, which were able to induce moDC maturation (71). Recently a study revealed that the interaction between CD155^+^ DCs and laquinimod-activated NK cells, which up regulated their DNAM-1 expression (recognizing CD155), led to suppression of experimental autoimmune encephalomyelitis (EAE). Interestingly, the immunoregulatory effect was not due to increased NK cell cytotoxicity against DCs, but rather due to induced reduction of HLA class II expression on DCs (72). Moreover, another group highlighted the role of the NK cell receptor NKG2D and its ligands during NK-DC interaction. They demonstrated that upon footpad injection of ectromelia virus, virus-infected murine skin-derived migratory DCs up regulated NKG2D ligands on their surface and migrated to the draining lymph nodes (dLN), where they stimulated IFNγ production by NK cells. IFNγ secretion then stimulated CXCL9 production in inflammatory monocytes resulting in an increased recruitment of circulating CXCR3^+^ NK cells to the dLN (73).

Interaction of NK cells and DCs was also suggested to control tissue-specific autoimmunity through an innate IFN-γ–IL-27 axis resulting in the generation if IL-10-producing Tr1-like cells (74) and to promote the tumor surveillance of other immune cells, a process described as the NK cell helper function. Activated human NK cells play a role in the induction of cDC1, which are producers of high amounts of IL-12p70. In this process, CD4^+^ T cells are primed to produce increased amounts of IFN-γ and less IL-4 favoring the induction of antigen-specific CD8^+^ T cells (75). Furthermore, mouse NK cells, recruited via CXCR3 into local lymph nodes, provide an early IFN-γ source to induce a Th1 immune response (76). In accordance with this report, only stimuli associated with the Th1 response are able to induce IFN-γ production in mouse NK cells, but not those, which are associated with the Th2 response (77).

## NK-DC Interaction Within the TME

The TME is a complex network comprising T reg cells, tumor-associated macrophages (TAMs), regulatory gamma-delta cells, myeloid-derived suppressor cells (MDSCs), soluble factors, the extracellular matrix and suppressive molecules expressed on tumor cells (78).

**Figure 1:**
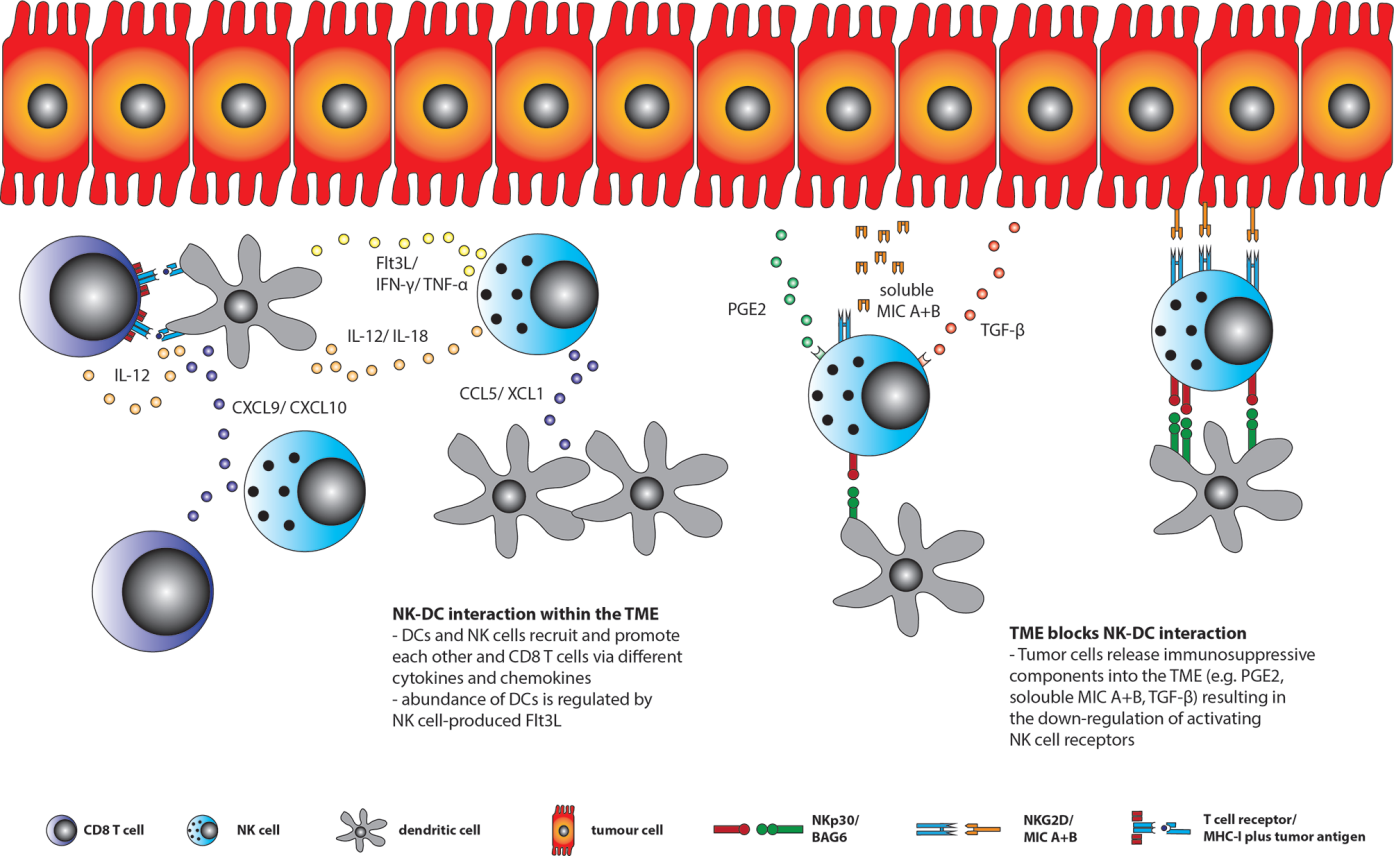
DC, CD8^+^ T and NK cell interaction within the TME. DCs, CD8^+^ T cells and NK cells may recruit each other into the TME by secreting various kinds of chemokines, like CCL5/XCL1 by NK cells to recruit DCs or CXCL9/10 by DCs to recruit NK and CD8^+^ T cells. Moreover, DCs activates CD8^+^ T cells via tumor antigen presentation and IL-12 production as well as TNFα and IFNγ secretion by NK cells through their own production of IL-12 and IL-18. In return, NK cell derived TNFα and IFNγ induce DC maturation. In addition, NK cells are the major source of the DC growth factor Flt3L. However, tumor cells are able to secret different compounds into the TME, like TGF-ß, soluble MIC A+B or PGE_2_, which are all able to down-regulate activating NK cell receptors (e.g. NKG2D and NKp30) resulting in reduced NK cell function and DC-NK cell interaction within the TME.

The interaction between DCs, NK cells and CD8^+^ T cells within the TME is diverse (**Figure 1**). Here, it was shown that the maturation of DCs is depending on NK cell derived HMGB1. Further, the chemokines CCL5 and XCL1/2 mediate the recruitment of cDCs to the TME, while the NK cell derived growth factor Flt3L ensures their survival. In return, IL-15 and IL-18 released by DCs active NK cells whereas DCs derived chemokines like CXCL9 and CXCL10 recruits NK cells and CD8^+^ T cells into the TME. Moreover, NK cells’ cytotoxic abilities and cytokine production, especially IFN-γ, is enhanced by cDCs in a cell-contact dependent and independent way (79, 80).

In a murine model, Allen et al. showed that tumor-derived CCL3 contributes to attract NK cells into the TME, leading to IFN-γ, DCs accumulation and T-cell recruitment (81). Also, in mouse, Wculek et al. demonstrated the capacity of cDCs bearing dead tumor cell antigens to highly induce CD8^+^ T cell response (82).

cDC1 express different chemokine receptors on their surface including the only receptor for XCL1, XCR1 (35) as well as receptors for CCL5 including CCR1 and CCR5 (83). Bottcher et al. demonstrated a reduced abundancy of cDC1, within tumors, when CCL5 and XCL1 was blocked in mice *in vivo*. Their data indicated that NK cell derived CCL5 and XCL1 recruits cDC1 into the TME of mouse tumors (4).

The localization of DCs within tumors is not only due to NK cell-derived chemokines but also depends on the local abundancy of growth factors like Flt3L. By depleting NK cells in tumor-bearing mice Barry et al. demonstrated that NK cell derived Flt3L regulates the amount of DCs within the tumor. These findings were further supported by the establishment of DC-NK cell conjugates within the TME (84).

It is well known that the TME induces tolerance and immunosuppression via various mechanism by dampening the functional activity of various immune cells, including NK cells and DCs (3). The TME alter the activation and cytokines production of pDCs and these pDCs are involved in tumor growth (85). Breast cancer or melanoma cells may reduce the expression of TLR or engage ILT7 (immunoglobulin like transcript 7) on pDC_s_ due to their BST2 expression leading to reduced IFN-α productions and release. They further induce the production of immunosuppressive TGF-β, resulting in reduced expression of activating NK cell receptors, including NKp30 and NKG2D, but not NKp46. Reduced NKp30 expression decrease NK cell cytotoxicity against immature DCs, subsequently leading to the accumulation of immature immunosuppressive DCs, probably supporting tumor growth. The release of soluble NKG2D ligands from tumor cells has been described to cause reduced expression of NKG2D on NK cells, reducing NK-DC-interaction upon IFN-stimulation. Moreover, data indicated that lower NKG2D levels on NK cells not only decreased their cytotoxic activity against NKG2D ligand-expressing tumor cells but also negatively influence IFN-α-mediated NK-DC-interactions, since NKG2D ligands are up regulated upon IFN-α stimulation on DCs. In addition, PGE_2_ was demonstrated to reduce NKG2D and 2B4 expression on NK cells, which not only inhibits IFN-γ production and cytotoxicity, but potentially effect NK-DC-interactions as well. When DCs are matured with PGE_2_s they were unable to recruit NK cells and were less effective in inducing IFN-γ production in NK cells. Tumor cell induced T reg cells inhibit the IL-15Rα-expression on DCs and thereby play a role in preventing the formation of a NK-DC immuno-synapse. The release of PGE_2_ by tumor cells was suggested to inhibit IL-18-secretion by DCs, which was associated with a reduction of HMGB1-production by NK cells leading to reduced DC maturation (3, 86).

## Discussion

**Figure 2:**
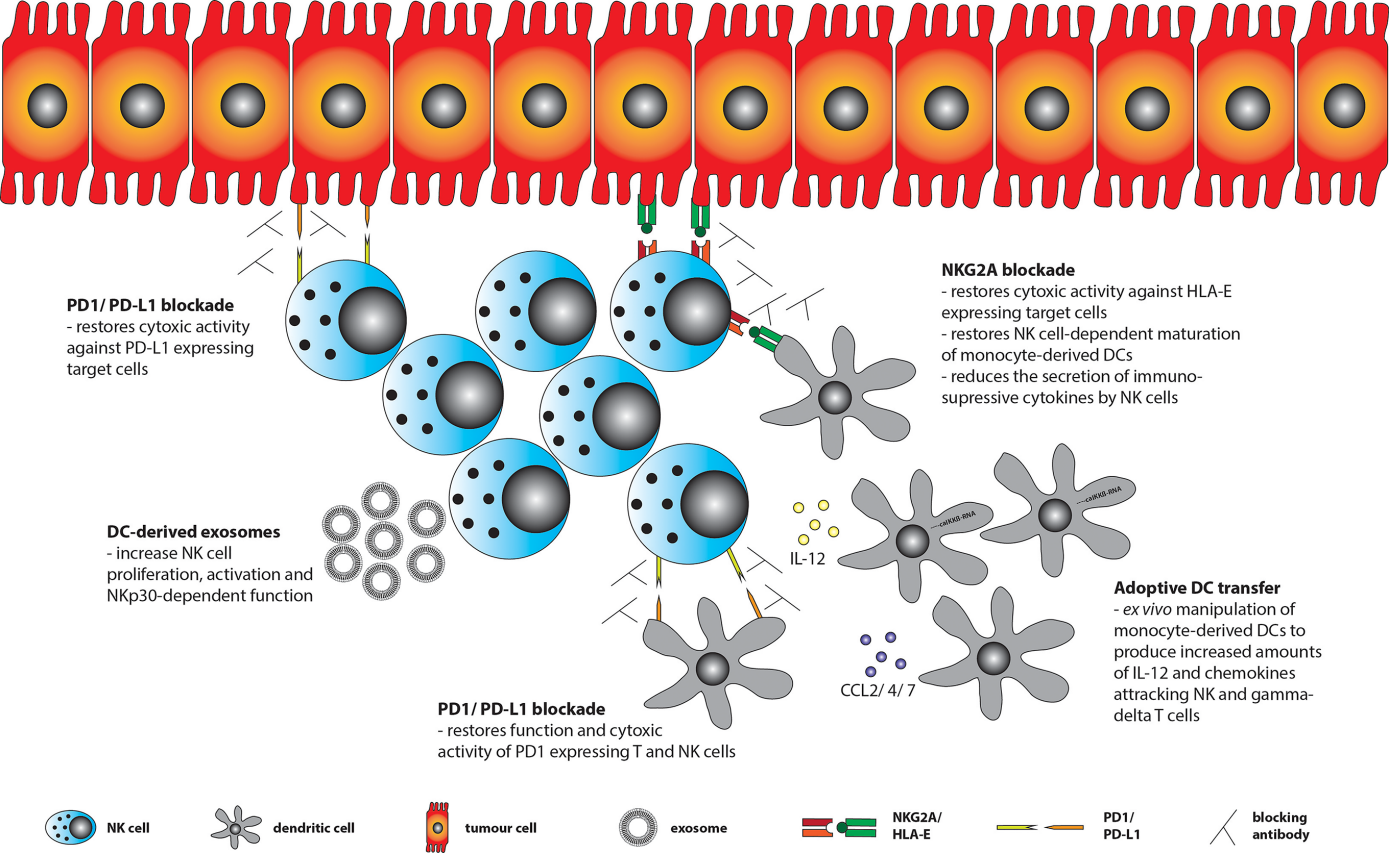
Treatment strategies to restore anti-tumor surveillance within the TME. Tumor cells can express ligands for various immune checkpoint (ICP) receptors including HLA-E (ligand for NKG2A) and PD-L1 (ligand for PD1), of which the latter one may also be expressed by tumor-infiltrating DCs. Using blocking antibodies against ICP receptors or their ligands restores NK cell function and DC-NK cell interaction. NK cells may further be stimulated by *ex vivo* generated DC-derived exosomes (Dex). In addition, adoptive transfer of *ex vivo* generated and engineered DCs (e.g. by transfecting them with a constitutively active IKKß (caIKKß) of the NF-kB pathway to enhance IL-12 production) could improve DC-NK cell interaction and eventually lift immunosuppression within the TME.

As pointed out the interaction between NK cells and DCs is negatively influenced by the TME in various ways. Therefore, strategies are needed to overcome these obstacles in order to restore anti-tumor surveillance within the TME (**Figure 2**).

### Optimization of DC Vaccination Products

One approach to optimize DC vaccination products might be to generate large amounts of pro-inflammatory DCs *ex vivo*, pulsed with tumor antigens, to lift immunosuppression within the TME. Until today various clinical trials using different DC products to treat cancer patients have been performed. Despite encouraging clinical responses in glioblastoma, pancreatic and prostate tumor patients (87–89), only sipuleucel-T has been approved by the FDA for the treatment of metastatic hormone refractory prostate cancer so far (90). This demonstrates that further improvement of the DC products is needed. For example, many trials used moDCs, which are quite convenient to be generated *in vitro*, but have the disadvantage, that they do not secrete IL12p70, when matured with a standard DC maturation cocktail (91, 92). This could be overcome by using mRNA electroporation to transfect moDCs with a caIKKß-RNA (constitutively active IKKß of the NF-kB pathway) enabling the transfected cells to enhance cytotoxic activity and IFN-γ production of autologous NK cells (92, 93). Another group used mRNA electroporation to engineer moDCs to produce IL-15/IL-15Rα or IFN-α leading to increased NK cell activation and cytotoxic activity against Burkitt lymphoma cells *in vitro* (94, 95). Increased NK cell cytotoxicity *in vitro* against Burkitt lymphoma cells was also achieved by co-culturing human NK cells together with TLR-activated CD1c^+^ myeloid and plasmacytoid DCs together instead of culturing them with only one specific DC subset alone (96). An additional approach is to optimize the DCs’ capacity to attract other immune cells, such as NK cells. Here, it would be interesting to understand the influence of IL-15 addition in the generation of *ex vivo* moDCs instead of IL-4. While IL-4 generated DCs secrete Th2 and T reg cell attracting chemokines such as CCL17 und CCL22, IL-15 rather induces the secretion of chemokines such as CCL2, CCL4, CCL7, CXCL9, CXCL10 and CXCL11, with CCL4 being the most important one to recruit NK and γδ T cells (97). Of note, IL-15 derived moDCs induced a stronger NK cell activation than IL-4 generated ones did (98). Moreover, IL-15 generated moDCs, that were matured with a human papilloma virus (HPV) vaccine, demonstrated a Th1-polarized cytokine profile, and increased the cytotoxicity of NK cells against cervical cancer cell lines (99).

### DC-Derived Exosomes a Vaccination Product

Nevertheless, certain obstacles remain when using DC vaccination with the aim to modulate the TME. DCs are still under the influence of the immunosuppressive milieu of the TME, need chemotactic signaling to reach their destination and a high amount of work is needed to produce and properly store them. One solution to overcome these obstacles could be the use of DC-derived exosomes (so called Dex) instead of whole DCs. Exosomes are nano-sized membrane vesicles, which can be secreted by various cell types. These exosomes are not only coated with HLA and costimulatory molecules on their surface, they also contain cytosolic proteins such as heat shock proteins and in addition mRNAs and small RNAs (100, 101). DC-derived exosomes have already been used within clinical phase I/II trials (102–104). Within an initial phase 1 trial, 15 melanoma patients were treated with DC-derived exosomes from tumor-antigen pulsed moDCs (102). A subsequent analysis demonstrated that the used DC-derived exosomes were able to stimulate proliferation and activation of NK cells *in vitro*, due to their expression of NKG2D ligands and IL-15Rα. Moreover, DC-derived exosomes treatment increased the absolute number of NK cells *in vivo* and restored NKG2D expression levels as well as K562-specific cytotoxicity in 7 out of 14 melanoma patients (105). Similar results were observed during a phase II trial treating inoperable non-small cell lung cancer (NSCLC) patients with DC-derived exosomes from IFNγ-matured moDCs as maintenance therapy after induction chemotherapy. Here, an increase of NKp30-dependent NK cell function was observed, although NKp30 expression itself was not increased on patients’ NK cells. Interestingly, NKp30-dependent function was associated with longer progression-free survival and with BAG6 expression, the ligand for NKp30, on the final DC-derived exosomes product (104). Taken together, DC-derived exosomes have the potential to induce NK cell proliferation and activation *in vivo* and *in vitro*. In addition, they have the unique property to resist the immunosuppressive milieu of the TME and to easily reach various compartments without the need of chemokine-dependent recruitment, making them an interesting option to manipulate NK-DC interaction within the TME.

### Blockade of Immune Checkpoint (ICP) Receptors

One of the major breakthrough in the treatment of tumor patients during the last decade was the introduction of blocking antibodies against immune checkpoint (ICP) receptors, especially against CTLA-4 and PD1, leading to long-term tumor control in melanoma, NSCLC and renal cell carcinoma patients. An important role for ICP receptors during the interaction between NK cells and DCs has been described for PD1, NKG2A and TIGIT.

PD-L1, the ligand for the ICP receptor PD1, is expressed on murine cDC1 and cDC2 (106) as well as on tumor and peripheral cDC and pDC of lung cancer patients (107). Its expression may limit the efficacy of DC vaccination trials, since PD-L1 inhibits proliferation and cytotoxic activity of PD1^+^ T and NK cells, respectively (108). The importance of PD1 expression on NK cells for tumor surveillance was demonstrated within the RMA-S lymphoma mouse model, which rather depends on NK cells controlling tumor growth than T cells. PD1 was strongly upregulated on NK cells within the TME and in draining lymph nodes. PD1 blockade led to a significantly reduced rate of tumor progression, which was not observed when NK cells were previously depleted. Interestingly, only a fraction of the NK cells expressed PD1. These cells were mostly activated NK cells and demonstrated higher functional activity than PD1 negative ones (109). PD1 expression on NK cells has been demonstrated as well in other tumor entities like Kaposi sarcoma (110), digestive cancers (111) and multiple myeloma (112). Using GM-CSF/IL-4 bone marrow-derived DCs (BMDCs) for vaccination in combination with pomalidomide and PD-L1 blockade, a significant tumor growth inhibition within a multiple myeloma mouse model could be achieved (113). Similar results were reported when using vaccination with GM-CSF/IL-4-derived BMDCs together with lenalidomide and PD1 blockade, which resulted in an increased functional activity of T and NK cells as well as a reduction of immunosuppressive cytokines within the TME (114). In addition, pDCs derived from the bone marrow of multiple myeloma patients expressed increased levels of PD-L1 and *in vitro* blockade of PD1 was able to enhance T cell proliferation and NK cell cytotoxicity during co-culture with myeloma-derived pDCs (115).

NKG2A is one of the major ICP receptors on NK cells recognizing the non-classical HLA molecule E (HLA-E) resulting in reduced cytotoxic activity against HLA-E positive target cells. MoDCs are able to upregulate NKG2A expression on NK cells in an IL12p70-dependent manner (116, 117). In addition, upon interaction with HLA-E expressing cells, NKG2A^+^ NK cells secreted increased amounts of IL-10 and TGF-ß resulting in reduced activation of moDCs and DC-mediated induction of CD4^+^CD25^+^ T reg cells. However, blockade of NKG2A was able to restore the activation of moDCs (118, 119). Currently, various clinical trials are testing NKG2A blockade within tumor patients. The first published and completed phase I/II trial in patients with advanced gynecologic malignancies demonstrated that the treatment was well tolerated, but only achieved a stable disease as maximal response (120). However, in patients with refractory/recurrent squamous cell carcinoma of the head and neck, the combination of an anti-NKG2A blocking antibody (monalizumab) together with an approved anti-EGFR antibody (cetuximab) demonstrated a confirmed RECIST (Response Evaluation Criteria in Solid Tumors) partial response in 8 of 26 (31%) and a stable disease in 14 of 26 (54%) patients during the interim’s analysis (121).

In addition, TIGIT is an inhibitory receptor recognizing the poliovirus receptor (PVR, CD155), such as the activating NK cell receptor DNAM1, but with a higher affinity. Importantly, binding of CD155 on moDCs by TIGIT^+^ T cells resulted in increased IL-10 and reduced IL-12p40 production (122). Interestingly, Flt3L-derived BMDCs derived from CD155^-^/^-^ mice were more sensitive towards NK cell killing than Flt3L-derived BMDCs from WT mice indicating a potential role for the CD155-TIGIT signaling pathway during the interaction of NK cells and DCs (123). Currently, various clinical trials are testing the potential of TIGIT blockade in tumor patients.

## Conclusion and Outlook

We have delineated that functions of DCs and NK cells as well as their interaction with each other are severely compromised within the TME. For this reason, various strategies to counteract immunosuppression within the TME have been discussed here. Although only PD1-PD-L1 blockade and sipuleucel-T have entered daily clinical life until today, further promising treatment options are currently being tested within clinical trials. Importantly, due to the complex and multiple facets of the TME, a combination of distinct treatment approaches will be inevitable to successfully lift immunosuppression from the TME without causing an immediate counter measurement by the tumor.

## Author Contributions

BJ and EU designed and coordinated the review. BJ, EU, VGe, LH, VGr, HS, and DD wrote the manuscript. All authors agree to be accountable for the content of the work. All authors contributed to the article and approved the submitted version.

## Funding

The laboratory of EU has been supported by the Frankfurt Cancer Institute FCI/DKTK (to EU), by the German Research Foundation DFG (CRC 1292, UL316/5-1), by the German Cancer Aid, the “Alfred & Angelika Gutermuth-Stiftung” and by “Menschen für Kinder e.V.”. HS was supported by the DFG (CRC 1292). DD was supported by funding from the German Research Foundation DFG (DU548/5-1, TRR305/1 project B05) and Agency national research (ANR)/German Research Foundation program (DU548/6-1) as well as intramural funds by the IZKF (A80).

## Conflict of Interest

The authors declare that the research was conducted in the absence of any commercial or financial relationships that could be construed as a potential conflict of interest.
